# A Haplotype-Resolved Genome Assembly of the Long-Spined Sea Urchin *Diadema antillarum*, a Keystone Caribbean Reef Herbivore

**DOI:** 10.3390/genes17080876

**Published:** 2026-07-28

**Authors:** Audrey J. Majeske, Juliet M. Wong, Carlos A. Farkas Pool, Jose M. Eirin-Lopez, Jose V. Lopez, Walter Wolfsberger, Nikolaos V. Schizas, Alondra M. Díaz-Lameiro, Stephanie O. Castro-Márquez, Kenneth Hilkert, Alejandro J. Mercado Capote, Taras K. Oleksyk

**Affiliations:** 1 Department of Biological Sciences, Oakland University, Rochester, MI 48309, USA; amajeske@oakland.edu (A.J.M.); wwolfsberger@oakland.edu (W.W.); stephaniecastro@oakland.edu (S.O.C.-M.); kchilkert@oakland.edu (K.H.); 2 Department of Biology, University of Puerto Rico at Mayagüez, Mayagüez, PR 00680, USA; alondra.diaz@upr.edu (A.M.D.-L.); alejandro.mercado1@upr.edu (A.J.M.C.); 3 Nicholas School of the Environment, Division of Marine Science and Conservation, Duke University Marine Lab, Beaufort, NC 28516, USA; juliet.wong@duke.edu; 4 Department of Biological Sciences, Florida International University, Miami, FL 33199, USA; jeirinlo@fiu.edu; 5 Laboratorio de Investigación en Ciencias Biomédicas, Departamento de Ciencias Básicas y Morfología, Facultad de Medicina, Universidad Católica de la Santísima Concepción, Concepción 4030000, Chile; cfarkas@ucsc.cl; 6 Department of Biological Sciences, Halmos College of Arts and Sciences, Nova Southeastern University, Dania Beach, FL 33004, USA; joslo@nova.edu; 7 Department of Marine Sciences, University of Puerto Rico at Mayagüez, Mayagüez, PR 00680, USA; nikolaos.schizas@upr.edu; 8 Department of Biology, Uzhhorod National University, 88000 Uzhhorod, Ukraine

**Keywords:** *D. antillarum*, *D. setosum*, sea urchin, genome assembly, haplotype-resolved, heterozygosity, echinoderm, conservation genomics, Caribbean reefs

## Abstract

**Background/Objectives**: The long-spined sea urchin *Diadema antillarum* is a keystone herbivore whose grazing maintains Caribbean coral reefs; basin-wide mass mortalities in 1983–1984 and 2022 have made genomic resources a conservation priority, yet no nuclear genome existed for the species. We aimed to generate the first nuclear reference and to resolve the high heterozygosity that complicates genome assembly in broadcast-spawning marine invertebrates. **Methods**: For the assembly, we combined PacBio HiFi, Oxford Nanopore, and Illumina sequencing. Genome size and heterozygosity were estimated by k-mer profiling. We compared standard and haplotype-aware assembly strategies (hifiasm), evaluated completeness with BUSCO, and annotated repeats using a species-specific RepeatModeler library. **Results**: k-mer profiling estimated a haploid genome of ~703 Mb with 2.52% heterozygosity. Standard assembly then produced an inflated 1.75 Gb assembly (98.4% BUSCO-complete but 84.4% duplicated), indicating retention of both haplotypes. Haplotype-aware reassembly separated this into a collapsed primary assembly (1.03 Gb) and two phased haplotypes (0.95 and 0.89 Gb), each comparable in size to the chromosome-level congener *D. antillarum* (886 Mb). BUSCO completeness reached 99.0%, with single-copy orthologs rising to 85–90%, and reference-free consensus quality reached QV 44.5 (Merqury; initial assembly). This genome is repeat-rich (42.84% repetitive; 29.96% unclassified). **Conclusions**: We provide the collapsed primary assembly together with both phased haplotypes as a haplotype-resolved reference for *D. antillarum*, establishing a foundation for immunogenomic, comparative, and population-genetic studies and for monitoring and restoration of this ecologically critical species. More broadly, the study shows that haplotype-aware assembly is essential for resolving such highly heterozygous genomes and delivers the genomic foundation needed to guide the conservation of this keystone Caribbean reef species.

## 1. Introduction

The long-spined black sea urchin *Diadema antillarum* (Philippi, 1845) is a keystone herbivore of Caribbean coral reefs. By grazing macroalgae, it maintains the open substrate that reef-building corals require to settle and grow, and its loss reshapes entire reef communities. The ecological weight of this single species was made starkly visible in 1983–1984, when a basin-wide mass mortality removed an estimated 93–100% of its numbers across the Caribbean and was followed by widespread shifts from coral- to algal-dominated reefs that showed little recovery over the following decades [1,2]. This species suffered a second mass mortality in 2022, this time linked to a parasitic ciliate closely matching *Philaster apodigitiformis* [3]. Such a comparable die-off, recurring nearly four decades after the first, renewed concerns that *D. antillarum* remains vulnerable to recurrent mortality events whose causes remain poorly understood to this day.

The recently reported complete mitochondrial genome was the first molecular resource of any kind for *D. antillarum* [4]. Beyond filling a conspicuous absence, with only a handful of partial gene sequences previously described for the species, it quickly demonstrated how genomic data can directly sharpen the systematics of this group. Specifically, using the four mitochondrial genomes available within Diadematidae at the time, it corrected inaccuracies in family-level phylogenetic relationships and identified a longstanding misidentification in the literature, where sequence data attributed to *Echinothrix diadema* most likely originated from *Diadema savignyi* [4]. Notably, that analysis did not recover *D. antillarum* and *D. setosum* as sister taxa, underscoring that relationships within the family are not yet fully resolved. Clearly, mitochondrial data alone provide limited resolution, and nuclear genomes would offer a far more comprehensive framework for comparative and phylogenetic analysis. Yet nuclear resources still remain scarce: although assemblies are now available for roughly two dozen echinoid species, only one exists within Diadematidae, the chromosome-level genome of the congener *D. setosum* [5]. This is unfortunate, as echinoid genomes have already proven their value, revealing unusually expanded innate-immune gene families: Toll-like receptors, NLR/NACHT-LRR receptors, scavenger receptor cysteine-rich proteins, and the rapidly evolving Trf/Sp185/333 family [6,7], gene systems of direct relevance to a species repeatedly struck by disease-driven mortality.

A nuclear reference for *D. antillarum* is a clear and pressing gap: it is the prerequisite for the immunogenomic, population-genetic, and comparative studies that will be required to understand and reverse this species’ decline. Assembling such a genome, however, is not straightforward. Like many broadcast-spawning marine invertebrates, *D. antillarum* is highly heterozygous, and high heterozygosity, particularly in combination with abundant repetitive DNA, is well known to impede genome assembly, thereby retaining the two parental haplotypes rather than collapsing them into a single haploid representation [8]. The same difficulty was encountered and explicitly documented during the assembly of *Strongylocentrotus purpuratus* [8]. Producing a usable reference for *D. antillarum* thus would depend not only on generating straightforward sequence data but also on resolving this heterozygosity during the assembly.

Here we present the first nuclear genome assembly for *D. antillarum*. Using long-read (PacBio HiFi, Oxford Nanopore) and short-read (Illumina) sequencing, we show that standard assembly retained both haplotypes, producing an inflated, duplicated assembly, and that haplotype-aware reassembly resolves this into a collapsed primary assembly with two phased haplotypes. We provide this haplotype-resolved assembly as a reference for *D. antillarum*, and characterize its size, completeness, repeat content, and heterozygosity. In doing so, we add the second nuclear genome to the family Diadematidae and establish a foundation for the comparative and conservation genomics that this ecologically critical, repeatedly imperiled species now urgently requires.

## 2. Materials and Methods

### 2.1. Sample Collection

**Figure 1:**
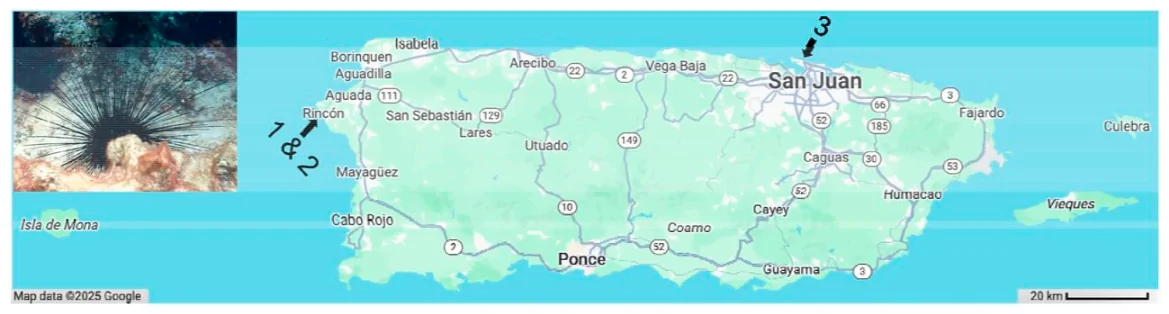
Map of Puerto Rico and collection sites of adult *D. antillarum*. Animals #1 and #2 were collected in Rincón (18°20′35.2″ N, 67°15′40.5″ W) in 2018; Animal #3 was collected at Punta Escambrón (18°27′59.70″ N, 66°05′10.26″ W) in 2022. Map modified from Google Maps (web version) (accessed on 14 February 2025). Inset: *D. antillarum* in its natural habitat (photo: A. J. Mercado Capote).

Two *D. antillarum* (An #1, An #2) adults were collected in Rincón, Puerto Rico, in 2018 (18°20′35.2″ N, 67°15′40.5″ W; Figure 1). For An #1, whole coelomic fluid (wCF) containing coelomocytes was withdrawn through the peristomal membrane using a sterile 23-gauge needle connected to a 5 mL syringe; the animal was photographed and returned to its habitat. Duplicate 1 mL aliquots were held on ice during transport to the University of Puerto Rico at Mayagüez (UPRM), where coelomocytes were pelleted by centrifugation, the cell-free fluid was replaced with RNAlater (Invitrogen, Carlsbad, CA, USA), and samples were frozen for shipment to Oakland University (OU). An #2 was transported live to UPRM, where Aristotle’s Lantern was dissected out and the animal inverted to decant ~15 mL of wCF; coelomocytes were aliquoted and processed as for An #1, stored at −20 °C, and shipped to OU.

A third animal (An #3) was collected by SCUBA at Punta Escambrón, Puerto Rico (18°27′59.70″ N, 66°05′10.26″ W; Figure 1) on 11 May 2022 and transported live to the University of Puerto Rico at Río Piedras, where it was spawned by intracoelomic injection of 0.53 M potassium chloride. Sperm was collected “dry” by pipetting directly from the test, rinsed three times in 1× phosphate-buffered saline (PBS), concentrated by centrifugation, frozen at −20 °C, and transported to Florida International University for DNA isolation.

The characteristics of the three specimens are summarized in Table 1. The reference assembly derives from a single individual (An #3); the two additional animals (An #1 and An #2) contributed only Oxford Nanopore reads used for gap-patching, so differences among the sampling sites do not affect the single-individual reference.

### 2.2. DNA Extraction, Library Preparation, and Sequencing

For the An #1 and An #2 coelomocyte samples, RNAlater was removed, and high-molecular-weight (HMW) DNA was extracted with a Monarch HMW DNA Extraction Kit (New England Biolabs, Ipswich, MA, USA); quality and quantity were assessed on an Implen C40 NanoPhotometer. Long-read libraries were prepared with the SQK-LSK109 ligation kit (Oxford Nanopore Technologies, ONT, Oxford, UK), quantified on a Qubit 2.0 fluorometer (dsDNA HS Assay; Thermo Fisher Scientific, Waltham, MA, USA), and sequenced across eight Flongle and four MinION flow cells (R9.4.1 or R10.4.1) on a MinION system (An #1, An #2; SRA experiments SRX27793263 and SRX27778283).

For the An #3 sperm sample (isolate JulietUrchin1), DNA was extracted with an E.Z.N.A. DNA/RNA Isolation Kit (Omega Bio-Tek, Norcross, GA, USA) and quality-controlled on a NanoVue Plus spectrophotometer (GE), a Qubit 4 fluorometer (Invitrogen), and a TapeStation (Agilent, Santa Clara, CA, USA). Genomic DNA was submitted to the University of Florida Interdisciplinary Center for Biotechnology Research (UF ICBR) for SMRTbell library construction and PacBio HiFi sequencing on a Sequel IIe SMRT Cell (diffusion loading, 2 h pre-extension, on-instrument HiFi read generation, 30 h movies), yielding 19.6 Gb of HiFi data (2,878,999 reads; ~28× coverage; SRA run SRR32365430). An aliquot of the same sperm DNA was used for Illumina library construction (New England Biolabs) and sequenced on an Illumina MiSeq (2 × 150 cycles) at UF ICBR (SRA experiment SRX27792652). In all cases, the extracted DNA passed instrument quality control and met the quantity and integrity requirements for library construction and sequencing; specific per-sample readings were not all retained in the project records, so the quality-control instruments and outcome are reported here in place of reconstructed values.

### 2.3. Read Processing and Contamination Screening

ONT reads were base-called with Guppy (v6.1.5), including adapter removal and retention of reads with Phred quality > 30; runs were combined per animal prior to downstream use. Illumina reads were quality-checked with FastQC (v0.12.1) and adapter-trimmed with Trimmomatic (v0.41). PacBio HiFi adapters were removed with HiFiAdapterFilt (v3.0.0) [9]; no HiFi reads were removed. To screen for foreign DNA, all read sets were checked for other diploid or prokaryotic sequences using BLAST+ (v2.16.0) [10]. Read sets were deposited at NCBI (Table 2).

### 2.4. Genome Profiling

Genome size, heterozygosity, and repetitiveness were estimated from adapter-free HiFi reads by counting k-mers (k = 21) with Jellyfish (v2.3.1) and modeling the spectrum with GenomeScope2.0 [11], which estimated a haploid genome of approximately 703 Mb and a heterozygosity of 2.52%. Ploidy was assessed with Smudgeplot (v0.2.6) using k-mer counts (k = 21 and 31) generated with FastK (v1.1); the result was consistent with a diploid genome.

### 2.5. Genome Assembly

To maximize assembly quality, we compared several pipelines (Table 3), in all cases using hifiasm (v0.19.8) [12] in its default mode, which outputs a single primary assembly. In the first pipeline, HiFi reads from the An #3 sperm sample were assembled with no scaffolding, polishing, or error correction, serving as a baseline. In the second, HiFi and ONT reads were assembled simultaneously without further refinement. In the third, RagTag (v2.1.0) [13] was used to order and orient the HiFi contigs using an ONT-based hifiasm assembly as a structural reference. In the final pipeline, HiFi reads were assembled with hifiasm, gaps and missing regions were patched with RagTag using Oxford Nanopore reads from two additional conspecific individuals (An #1 and An #2), and the assembly was polished with Illumina short reads from the same sperm individual used for the HiFi assembly (An #3) using Racon [14]. No classical scaffolding was performed; RagTag was used for patching only. Because the Nanopore patching reads derived from individuals other than the HiFi source, their contribution was deliberately limited to gap-patching, whereas base-level polishing used the same-individual Illumina data. The resulting assembly (GCA_040938485.1) spanned 1.75 Gb (1,754,659,819 bp) across 2964 contigs with an N50 of 1.57 Mb and contained no gaps (total gap length = 0), indicating that the cross-individual patching introduced no scaffold-level sequence and that the reference rests on single-individual HiFi data.

This primary assembly showed signs of incomplete haplotype collapse: BUSCO completeness (metazoa_odb10) [15] was high, but the large majority of complete orthologs were duplicated rather than single-copy, and the assembly was roughly twice the read-based size estimate. Given the species’ high heterozygosity, this pattern is consistent with both haplotypes being retained in the default primary output. Applying Purge_Dups (v1.2.5; see Section 2.8) [16] to this assembly reduced but did not eliminate ortholog duplication and lowered overall completeness (Table 4), indicating that post hoc redundancy removal alone did not fully separate the haplotypes.

### 2.6. Haplotype-Resolved Assembly

To separate the two haplotypes directly, HiFi reads were reassembled with hifiasm (v0.19.8) in haplotype-aware mode (hifiasm *—dual-scaf*), yielding a collapsed primary assembly and two phased haploid assemblies (hap1, hap2). Because the reference derives from a single wild-caught individual (An #3) and no parental samples were available, trio-based phasing was not possible; haplotypes were therefore resolved from the HiFi reads alone. No Hi-C or other long-range scaffolding data were generated; consequently, no chromosome-scale scaffolding was performed, and the assembly is reported at the contig level. The collapsed primary was then polished with trimmed Illumina paired-end reads over two rounds of Racon (v1.5.0) [14], with reads mapped to the assembly using Minimap2 (v2.28, *-ax sr* preset) [17] and sorted with SAMtools (v1.21) [18]; no RagTag patching was applied to this assembly. Assembly sizes, contig counts, and N50 values were taken directly from the assembled sequences: the collapsed primary (Racon-polished) spanned 1,028,719,238 bp (1.03 Gb; 2051 contigs; N50 1.20 Mb), and the two phased haplotypes, hap1 and hap2, spanned 946,558,832 bp (0.95 Gb; 2860 contigs; N50 662 kb) and 892,201,912 bp (0.89 Gb; 2400 contigs; N50 682 kb), respectively. The Racon-polished collapsed primary together with the two phased haplotypes constitutes the haplotype-resolved reference reported here.

### 2.7. Assembly Evaluation and Completeness

Contiguity metrics (total length, contig number, N50) were assessed with QUAST (v5.2.0, reference-free) [19] and Bandage (v0.8.1) [20], and overall assembly characteristics were visualized as a snail plot with BlobToolKit (v3.0.6) [21]. Genome completeness was evaluated with BUSCO (v5.3.0) [15]. The v0.4 assembly and its Purge_Dups output were assessed against the metazoa_odb10 lineage set; the haplotype-aware assemblies (collapsed primary and the two haplotypes) were assessed against metazoa_odb12. All BUSCO scores are reported together with their corresponding lineage-set version (Table 4). Reference-free consensus quality (QV) and k-mer completeness were additionally estimated with Merqury (v1.4.1) [22], using a hybrid meryl (v1.4.1) k-mer database (k = 21) built from the PacBio HiFi and Illumina reads; the resulting copy-number spectrum is shown in Supplementary Figure S4 and the values are reported in Section 3.1.

### 2.8. Characterization of the Initial Assembly

The following analyses characterize the initial (v0.4) assembly and the nature of its retained duplication; they are reported as supporting observations. Raw HiFi reads were mapped back to the assembly with Minimap2 (v2.28) [17] and processed with SAMtools (v1.21) [18]; per-base coverage statistics were computed with GNU Awk (v5.1.0) and plotted with Matplotlib (v3.10.1). Reads exceeding 100× and those above three times the modal 11× coverage (i.e., >33×) were extracted for further inspection. Large structural variants were called from HiFi reads with Sniffles (v2.6.0) [23] and retained at a minimum length of 1000 bp and classified by type (deletions, insertions, duplications, inversions, and breakends). Repeats were characterized by building a custom *D. antillarum* repeat library with RepeatModeler (v2.0.3) and annotating the assembly with RepeatMasker (v4.1.5), yielding 42.84% repetitive content (29.96% unclassified). Contig redundancy was assessed with Purge_Dups (v1.2.5) [16] using coverage-derived cutoffs (5, 9, 15, 18, 30, 54); redundancy statistics between the retained and purged outputs were computed with a custom Python script (Zenodo: 10.5281/zenodo.15172401).

### 2.9. Comparative Analysis

Assembly size, contiguity, heterozygosity, repeat content, and gene number were compared with those of the congener *D. setosum*, the only other chromosome-level nuclear genome available within the family (Supplementary Table S2).

### 2.10. Genome Annotation Pipeline

The assembly was annotated by the NCBI Eukaryotic Genome Annotation Pipeline (EGAP v10.3; annotation release GCF_040938485.1-RS_2025_02). The pipeline incorporated 9 same-species *D. antillarum* GenBank transcripts (aligned with Splign at 98.93% average identity) and 12 *D. antillarum* RNA-Seq runs spanning coelomic fluid, body wall, gonad, and esophagus tissues (274.5 million reads in aggregate), aligned with STAR. The RNA-Seq evidence included data generated by our laboratory: two gonad Ion Torrent runs (SRR24973326, SRR24973327) and an esophagus Illumina run (SRR29948272), together accounting for the majority of the aligned reads. For evidence alignment, the genome was masked with WindowMasker (37.94% of the assembly), distinct from the RepeatMasker-based repeat annotation reported in Section 3.5. Transcript and protein alignments were integrated with ab initio models by Gnomon, yielding 33,123 protein-coding genes, 3091 non-coding transcripts, and 1544 pseudogenes.

## 3. Results

### 3.1. Sequencing and Genome Profiling

**Figure 2:**
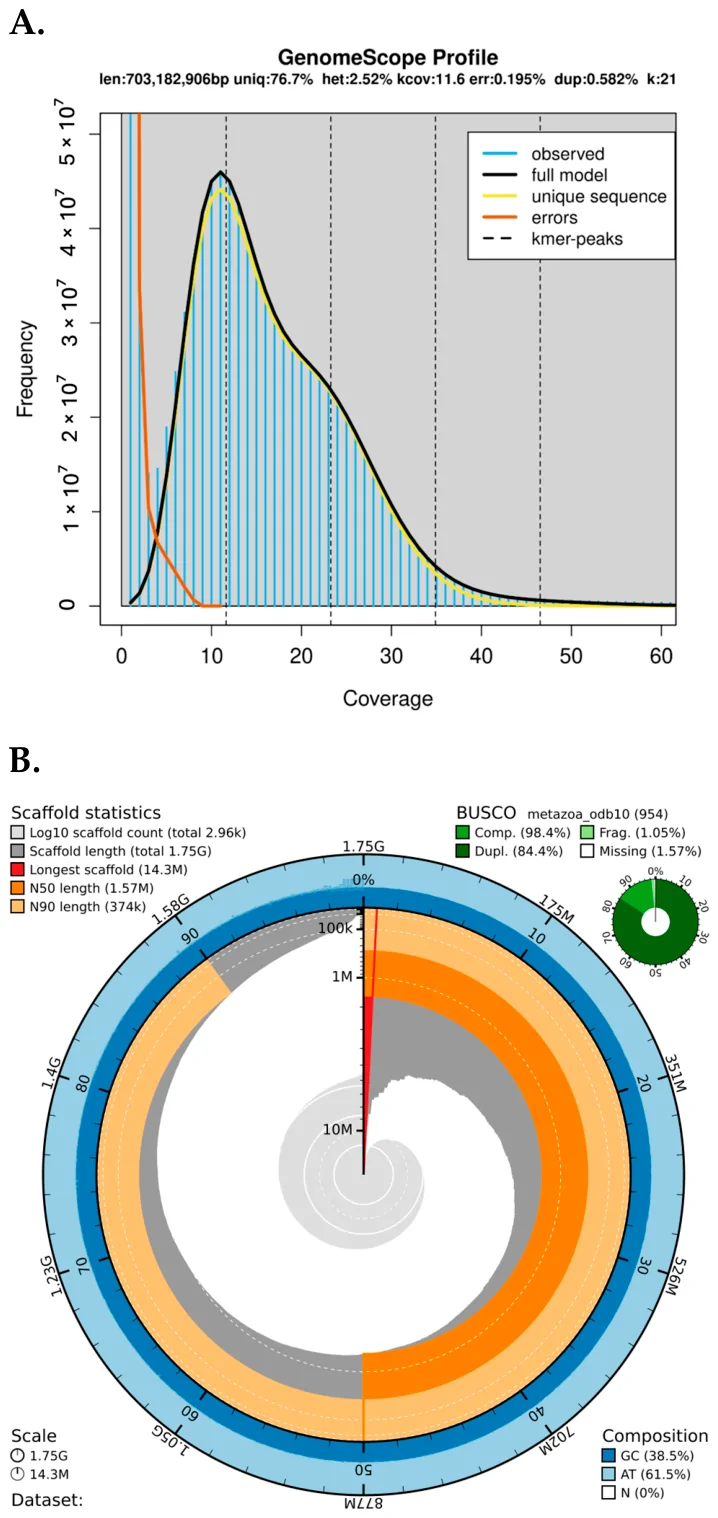
Genome profiling and initial assembly statistics for *D. antillarum*. (**A**) GenomeScope2.0 k-mer spectrum (k = 21) of adapter-free HiFi reads, modelling a heterozygous diploid genome (heterozygosity 2.52%; haploid length ≈ 703 Mb). The major peak near ~11× and the secondary peak near ~23× correspond to the heterozygous and homozygous k-mer fractions expected of a diploid genome at this heterozygosity. (**B**) BlobToolKit snail plot of the initial (uncollapsed) hifiasm v0.4 assembly (1.75 Gb); BUSCO completeness against metazoa_odb10 is dominated by duplicated orthologs (Comp. 98.4%, of which Dupl. 84.4%), reflecting the retained haplotypes resolved in Section 3.3.

PacBio HiFi sequencing of the An #3 sperm sample produced 2,878,999 reads totaling 19,633,500,452 bp, with a mean read length of 6819 bp, a mean read quality of Q34, and a mean of 13 sequencing passes. ONT and Illumina yields are summarized in Table 2. *k*-mer analysis of the adapter-free HiFi reads with GenomeScope2.0 predicted a haploid genome length of 703,182,906 bp, with 76.7% uniqueness, a heterozygosity of 2.52%, a duplication ratio of 0.582%, and an error rate of 0.195% (model fit 97.75 ± 1.17%; Figure 2A). Reference-free evaluation of the initial hifiasm assembly with Merqury (HiFi and Illumina k-mers) returned a consensus quality of QV 44.5—approximately one error per 28 kb (~99.996% base accuracy)—with 97.5% k-mer completeness (Supplementary Figure S4). Merqury QV could be computed directly on the collapsed primary and on each phased haplotype; however, for a highly heterozygous diploid, these values would be biased downward because a collapsed or single-haplotype representation omits one allele at every heterozygous site and the corresponding read k-mers then register as missing, artificially depressing both the QV and the k-mer completeness estimate. We therefore report QV on the initial assembly, which retains both haplotypes and thus captures the read k-mer content most completely, providing the most representative reference-free estimate of base-level accuracy for the HiFi and Illumina data shared by all assembly versions. Smudgeplot at *k* = 21 recovered four genotype configurations (AB, AAB, AAAB, AABB) at frequencies of 0.61, 0.23, 0.03, and 0.05; at *k* = 31, only AB and AAB were recovered (0.73 and 0.18; Supplementary Figure S1). These profiles are consistent with a diploid genome.

### 3.2. Initial Assembly and Ortholog Duplication

Among the assembly pipelines evaluated (Table 3), the hifiasm v0.4 pipeline produced the most contiguous initial assembly, spanning 1.75 Gb across 2964 contigs with an N50 of 1.57 Mb. BUSCO assessment of this assembly (metazoa_odb10) returned 98.4% completeness; however, only 14.0% of orthologs were single-copy, with 84.4% complete-and-duplicated (Table 4; Figure 2B). The assembled length was approximately 2.5× the GenomeScope haploid estimate (703 Mb) and roughly twice the size of the chromosome-level congener *D. setosum* (Supplementary Table S2).

### 3.3. Resolving the Duplicated Content

Applying Purge_Dups to the v0.4 assembly produced a “cleaned” assembly of 1.24 Gb in 1024 contigs, with the redundant sequences (2015 contigs, 519 Mb) removed to a separate haplotig set; BUSCO completeness was 97.4%, with single-copy orthologs increasing to 62.5% and duplicates falling to 34.9% (Table 4). This reduced but did not eliminate the duplication.

**Figure 3:**
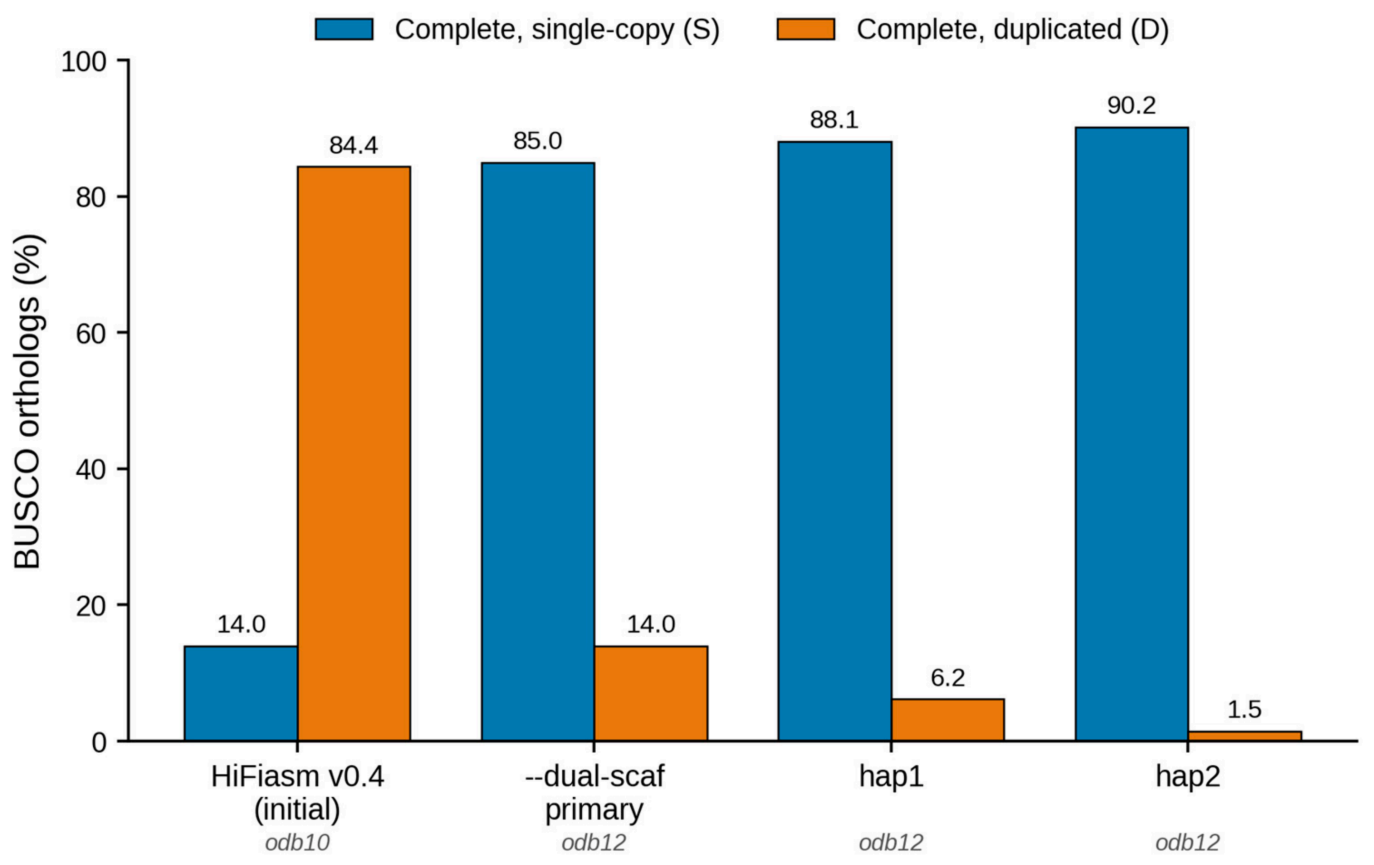
Resolution of ortholog duplication by haplotype-aware assembly. Grouped bars show complete single-copy (S) and complete duplicated (D) BUSCO percentages for the initial hifiasm v0.4 assembly and the —dual-scaf collapsed primary and phased haplotypes (hap1, hap2). Single-copy completeness rises from 14.0% in the initial assembly to 85.0% in the collapsed primary and 88.1–90.2% in the haplotypes, with duplication falling correspondingly. BUSCO lineage sets differ between the initial assembly (metazoa_odb10) and the haplotype-aware assemblies (metazoa_odb12), as indicated beneath each bar; the single-copy/duplicated ratio shift is robust to this difference.

Reassembly with hifiasm in haplotype-aware mode (*—dual-scaf*) resolved the duplication more completely. This produced a collapsed primary assembly of 1.03 Gb (1,028,719,238 bp after Racon polishing; 2051 contigs; N50 1.20 Mb) and two phased haplotype assemblies: hap1 (946,558,832 bp; 0.95 Gb; 2860 contigs; N50 662 kb) and hap2 (892,201,912 bp; 0.89 Gb; 2400 contigs; N50 682 kb). BUSCO completeness (metazoa_odb12) was 99.0% for the collapsed primary, with single-copy orthologs at 85.0% and duplicates at 14.0%; for the phased haplotypes, single-copy completeness rose to 88.1% (hap1) and 90.2% (hap2), with duplicates falling to 6.2% and 1.5%, respectively (Table 4; Figure 3).

### 3.4. The Haplotype-Resolved Reference Assembly

The two phased haplotypes each spanned approximately 0.9 Gb, comparable to the *D. setosum* assembly (885.8 Mb; Supplementary Table S2), and together approximated the ~1.75 Gb of the initial v0.4 assembly. We report the collapsed primary together with hap1 and hap2 as the haplotype-resolved reference for *D. antillarum* (see Data Availability Statement).

### 3.5. Repeat Content and Assembly Characterization

**Figure 4:**
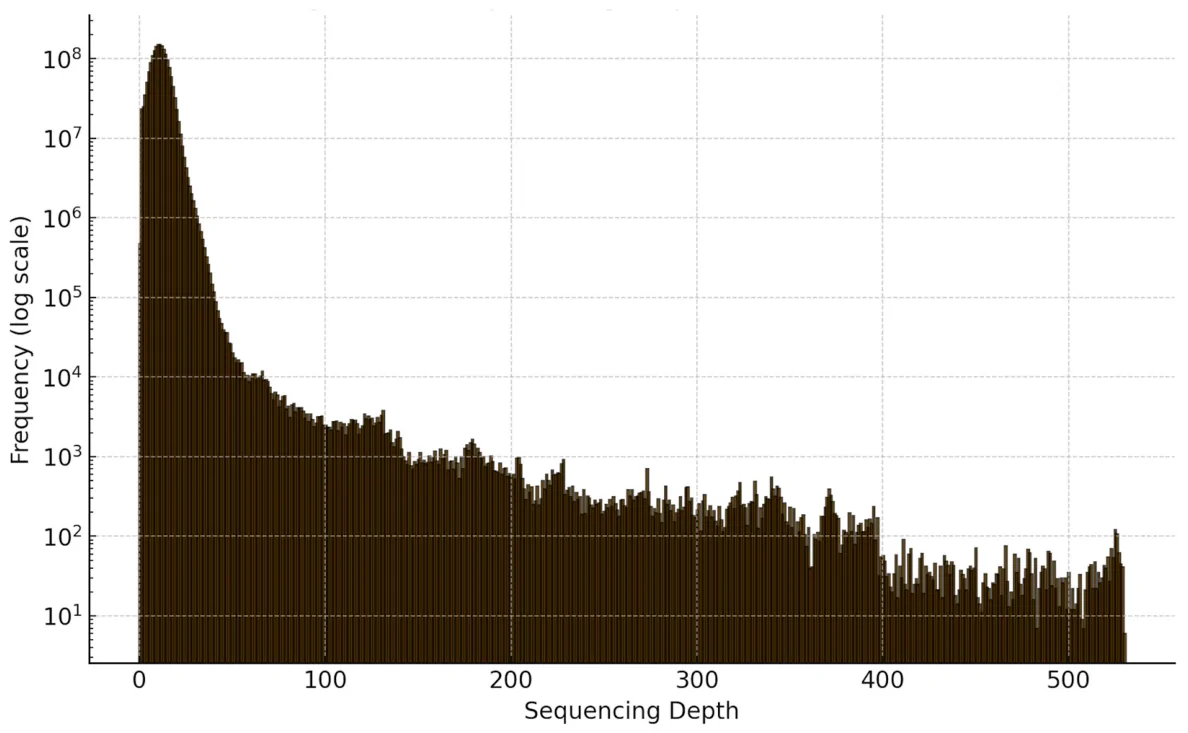
HiFi sequencing-depth distribution across the initial (uncollapsed) hifiasm v0.4 assembly. HiFi reads were mapped back with Minimap2 (v2.28) and per-base depth summarized with Matplotlib (v3.10.1) on a logarithmic frequency scale. The primary peak at ~11× represents the bulk of the assembly; a right-skewed tail extends to >530×, corresponding to high-copy and repetitive regions.

Repeat annotation of the v0.4 assembly with a custom RepeatModeler library identified 42.84% of the assembly as repetitive, of which 29.96% was unclassified (Supplementary Table S3). Mapping raw HiFi reads back to the v0.4 assembly produced a modal coverage of ~11×, with a long tail of higher-coverage regions extending to ~530× (Figure 4); reads exceeding three times the modal coverage (>33×) accounted for 30.53% of the repetitive content. These high-coverage reads were themselves progressively more repetitive: 61.52% of bases were masked as repeats in the >33× read set and 93.07% in the >100× set, compared with 42.84% across the assembly as a whole (Supplementary Table S3), confirming that the high-coverage tail is dominated by repetitive sequence.

Mapping the HiFi reads back to the assembly, Sniffles2 (v2.6.0) identified 2326 structural variants ≥ 1 kb—1289 deletions, 388 insertions, 34 duplications, 15 inversions, and 600 breakend calls. The deletions, insertions, and duplications span 9.3 Mb (0.53% of the assembly), with the 15 inversions adding a further 8.6 Mb (Supplementary Figure S5). These calls are overwhelmingly heterozygous and, being derived from a self-alignment of the reads to the assembly they built, are best interpreted as differences between the two retained haplotypes of this individual—and, for the breakend calls, as contig-fragmentation artifacts, rather than as external or population-level structural variation. Redundant mapping of raw reads to the assembly was 0.38%.

Taken together, these read-mapping metrics—a consistent modal depth with an elevated-coverage tail confined to repetitive sequence, a low redundant-mapping rate (0.38%), and structural variants that are predominantly heterozygous (consistent with retained haplotypic differences rather than misassembly)—provide read-based evidence for the structural integrity of the contig-level assembly; chromosome-scale validation would additionally require long-range (Hi-C) contact data, which were not generated here.

### 3.6. Genome Annotation

NCBI EGAP annotation of the v0.4 assembly identified 33,123 protein-coding genes, 3091 non-coding transcripts, and 1544 pseudogenes (accession GCF_040938485.1-RS_2025_02); this annotation incorporated the RNA-Seq evidence described in Section 2.10. Protein-coding genes had a mean genomic span of 24,325 bp; across all 36,214 annotated genes, the mean was 22,714 bp (22,659 bp including the 1544 pseudogenes). Genes averaged 9.00 unique exons each (8.63 including pseudogenes), while the 36,155 protein-coding transcripts averaged 9.45 exons per transcript (coding exons only; 10.02 across all exon features). These statistics derive from the annotation of the uncollapsed v0.4 assembly, which retains allelic duplicates (Section 4.5), and should be interpreted with that caveat.

For comparison, the *D. setosum* assembly contains approximately 23,000 protein-coding genes (Supplementary Table S2). Assessed against the same metazoan BUSCO set (metazoa_odb10, n = 954), the two assemblies recover almost identical fractions of the conserved gene space: 98.4% complete (0.6% missing) for *D. antillarum* and 98.1% complete (0.7% missing) for *D. setosum*. These values indicate near-complete overlap of the core gene content in both species, with under 1% missing in each, and at least ~96% of these orthologs are complete in both.

## 4. Discussion

We present the first haplotype-resolved nuclear genome assembly for *D. antillarum*, comprising a collapsed primary assembly (1.03 Gb) and two phased haplotypes (hap1, 0.95 Gb; hap2, 0.89 Gb). This resource adds the second nuclear genome within the family Diadematidae, alongside the chromosome-level assembly of the congener *D. setosum*, and provides a framework for the comparative, immunogenomic, and population-genetic studies that the recent history of mass mortality in this species makes urgent.

### 4.1. The Expanded Initial Assembly Reflects Incomplete Haplotype Collapse

Our initial assembly (hifiasm v0.4) spanned 1.75 Gb: approximately 2.5× the GenomeScope haploid estimate of 703 Mb and roughly twice the size of *D. setosum* (886 Mb). Several lines of evidence indicate that this inflation was driven predominantly by the retention of both haplotypes in a single assembly rather than by a genuine expansion of the genome. The most direct evidence is the behavior of single-copy orthologs. In the v0.4 assembly, BUSCO completeness was high (98.4%), but it was overwhelmingly duplicated (84.4% duplicated, only 14.0% single-copy), a retained-haplotype signal also evident in the k-mer copy-number spectrum (Supplementary Figure S4). When the haplotypes were separated with haplotype-aware assembly (*—dual-scaf*), duplication collapsed: the primary assembly returned 85.0% single-copy orthologs, and the two phased haplotypes returned 88.1% and 90.2% single-copy, with duplication falling to 6.2% and 1.5%, respectively (Figure 4). A conserved single-copy gene appearing twice in the v0.4 assembly and once in each separated haplotype is the expected signature of two retained alleles, not of a genuinely duplicated locus.

This interpretation is reinforced by genome size. The two phased haplotypes each span approximately 0.9 Gb, close to the *D. setosum* assembly (886 Mb) and to the values expected for a single *Diadema* haploid genome, whereas a true doubling of the genome would leave each haplotype near the full 1.75 Gb. The diploid structure recovered by Smudgeplot, and the moderate heterozygosity of the species (2.52%), are likewise consistent with a heterozygous diploid whose alleles were incompletely collapsed under the default assembly mode.

These conclusions rest on several independent lines of evidence that converge on the same interpretation. The behavior of single-copy orthologs across assembly stages (Table 4), the concordance of the phased haplotype sizes with both the GenomeScope haploid estimate and the chromosome-level *D. setosum* genome, the diploid AB signal from Smudgeplot (Supplementary Figure S1), the pronounced copy-2 peak in the Merqury k-mer copy-number spectrum (Supplementary Figure S4), and the predominantly heterozygous structural-variant profile recovered by self-alignment (Supplementary Figure S5) each rest on different data and assumptions—ortholog presence, k-mer multiplicity, ploidy modeling, and read alignment—yet all indicate retained heterozygous haplotypes, rather than genuine genome expansion, as the dominant cause of the inflated initial assembly. Because these orthogonal approaches agree, the haplotype-collapse interpretation is robust, and the collapsed primary is well justified as the reference.

### 4.2. Residual Duplication and the Possibility of Genuine Repeat Expansion

The collapse interpretation does not exclude some genuine duplicated or repetitive content. The phased haplotypes (~0.9 Gb) remain somewhat larger than the GenomeScope haploid estimate (703 Mb), and hap1 retains 6.2% duplicated orthologs, indicating that haplotype separation was not complete. The assembly is also markedly repeat-rich (42.84% repetitive, of which 29.96% is unclassified), and repeat-copy-number differences between haplotypes are a well-documented cause of incomplete collapse [12,16]. A modest amount of real duplication or lineage-specific repeat expansion may therefore be superimposed on the dominant haplotype-collapse signal. Discriminating these contributions at the level of individual loci is beyond the resolution of the present assembly; we return to this point as a direction for future work (Section 4.6).

### 4.3. Heterozygosity in a Broad Comparative Context

The heterozygosity of *D. antillarum* (2.52%) is best understood in the context of the appropriate comparative baseline. Relative to most vertebrates, in which nucleotide diversity is typically on the order of 0.1%, a value of 2.52% is high—more than an order of magnitude greater. It is only “moderate” within echinoids and broadcast-spawning marine invertebrates, a group characterized by exceptionally high genetic diversity [24]: heterozygosity reaches 4–5% in the purple urchin *S. purpuratus* [8] and 4.4% in the sea urchin *Echinometra sp. EZ* [25], while broadcast-spawning mollusks span roughly 0.2–2.4% across species [26]. Against this background, *D. antillarum* (2.52%) and *D. setosum* (2.11%) sit toward the lower end of the echinoid range while remaining high in absolute terms.

This pattern has a well-established explanation. Broadcast-spawning marine invertebrates combine high fecundity, long-lived planktonic larvae, and few physical barriers to gene flow, which together sustain very large, genetically diverse populations. High reproductive variance (“sweepstakes” recruitment) can complicate the relationship between census and effective population size [27], but the net result for genome assembly is consistent: outbred marine invertebrates carry abundant allelic variation. Notably, the *S. purpuratus* genome project encountered the same difficulty reported here: heterozygosity high enough to obstruct the merging of homologous reads and to blur the distinction between heterozygous alleles and duplicated loci [8]. The collapse failure we observed under default assembly settings is therefore an expected consequence of assembling a highly heterozygous, repeat-rich echinoid genome, not an anomaly of this dataset.

The relatively lower heterozygosity of *D. antillarum* within the echinoid range also raises a conservation question. The Caribbean population retained high mitochondrial diversity and a signature of past population expansion despite the >97% mortality of 1983–84, based on ATPase mtDNA sequences [28]. Whether the more recent die-offs have left a detectable genome-wide signature is now testable with this assembly and is a natural direction for population-genomic follow-up.

### 4.4. Value of the Resource and Implications for Conservation

The value of this genome lies primarily in the analyses it enables for a species in urgent need of them. *D. antillarum* has experienced repeated mass mortalities: the basin-wide 1983–1984 event [2] and the 2022 die-off attributed to a scuticociliate most similar to *P. apodigitiformis* [3]—yet the molecular tools needed to understand and respond to these events have been lacking. Echinoid genomes have revealed unusually expanded and diversified innate-immune gene families, including Toll-like receptors, NLR/NACHT-LRR receptors, scavenger receptor cysteine-rich proteins, and the rapidly evolving Trf/Sp185/333 family [6,7]; a genome for *D. antillarum* enables immunogenomic investigation of these families and, in particular, tests of whether immune-gene variation is associated with survival during the disease-driven die-offs. Beyond immunology, the assembly supports genome-wide SNP development for range-wide population monitoring, and assessment of connectivity and source–sink dynamics to inform which populations reseed reefs after mortality and where restoration effort is best directed.

The single observation with immediate conservation bearing is the heterozygosity of the sequenced individual (2.52%), which is high for an animal and comparable to its congener *D. setosum*. However, a single genome cannot support population-level inference about diversity or bottlenecks; multi-individual sampling, now feasible against this reference, will be required to test whether recent mortality events have left a genome-wide signature (see Section 4.3). As the second nuclear genome within Diadematidae, alongside the chromosome-level assembly of *D. setosum*, the resource also establishes a basis for comparative and phylogeographic study across the family.

### 4.5. Evolutionary Context

As the second nuclear genome sequenced within Diadematidae, this assembly contributes primarily as comparative material for understanding echinoid genome evolution rather than as a source of evolutionary conclusions in itself. Two features are worth noting. First, the genome is markedly repeat-rich (42.84% repetitive, 29.96% unclassified), consistent with the large and incompletely characterized repeat fractions reported across echinoid genomes. Second, the species’ heterozygosity (2.52%) reflects the large effective population sizes typical of broadcast-spawning marine invertebrates (Section 4.3), placing *D. antillarum* within the high-diversity, assembly-challenging regime exemplified by *S. purpuratus* [8].

We caution against over-interpreting the gene count: the NCBI annotation (33,123 protein-coding genes) exceeds the estimates for *D. setosum* (~23,000) and *S. purpuratus* (~23,300), but because it was generated on the uncollapsed assembly, this difference could be an artifact of allelic duplication rather than evidence of genuine gene-family expansion, and cannot be interpreted evolutionarily until the collapsed reference is re-annotated. The annotated gene set itself carries the same signature: BUSCO assessment of the predicted proteins (metazoa_odb10) returned 99.3% complete but 86.9% duplicated, with only 12.4% single-copy, closely mirroring the 84.4% duplication of the underlying assembly. The gene models are therefore duplicated to the same degree as the assembly they were built on, independently confirming that the elevated gene count reflects retained haplotypes rather than true gene-family expansions.

Beyond comparative analysis, the assembly provides the reference against which targeted resequencing markers can be designed and population-scale data anchored: the practical step that converts a single genome into population-level inference. With a reference in hand, reduced-representation approaches (e.g., RAD/ddRAD), target-capture panels for specific gene sets, and reference-mapped whole-genome resequencing all become feasible across many individuals. Such panels would enable the population-level tests of selection on the echinoid immune-gene families noted in Section 4.4, once multi-individual data are available. The same framework extends to the ecological pressures of a warming Caribbean, allowing tests of whether variation in immune and thermal-tolerance genes tracks disease exposure or thermal regime, and whether the species harbors the standing variation needed to respond to ongoing environmental change. Finally, even from the single genome reported here, coalescent-based demographic reconstruction (e.g., PSMC) could begin to recover the historical population-size trajectory of the species, complementing the mitochondrial and allozyme records of its response to past mortality events [28]. Whole-genome alignment, synteny analysis, and gene-family comparison with *D. setosum* and other echinoids are the subject of ongoing work. Because collinearity, chromosomal rearrangement, and fusion/fission events can be assessed robustly only between chromosome-scale assemblies on both sides, these comparisons are not attempted at the present contig-level resolution and are deferred to future work.

### 4.6. Limitations and Future Work

Several limitations should be noted. The assembly is contig-level rather than chromosome-scale (no Hi-C or long-range scaffolding data were generated); haplotype separation is incomplete (hap1 retains 6.2% duplicated orthologs); and a substantial fraction of the repeat content remains unclassified. The genome was assembled from a single individual, so it does not capture population-level variation. Because no parental samples were available, trio-based phasing could not be performed, and haplotypes were resolved from HiFi data alone. Importantly, the NCBI EGAP annotation (33,123 protein-coding genes) was generated on the initial, uncollapsed v0.4 assembly; because gene models on an assembly retaining both haplotypes may be inflated by allelic duplication, the gene count relative to *D. setosum* (~23,000) should be interpreted with caution: the annotated gene set is itself 86.9% duplicated by BUSCO (Section 4.5), and re-annotation of the collapsed reference is a priority.

The haplotype-resolved assembly resolves the duplication that complicated the initial assembly, but the present data cannot, on their own, distinguish residual allelic redundancy from any genuine recent paralogy at the level of individual loci. On the other hand, the collapse interpretation does not exclude some genuine duplicated or repetitive content. A telomere-to-telomere, chromosome-scale assembly, achievable by adding long-range scaffolding (e.g., Hi-C) and ultra-long ONT reads (the latter to improve repeat resolution and the assembly of complex genomic regions) to the high-coverage long-read data generated here, would permit direct discrimination between these alternatives, by resolving whether duplicated sequences occupy allelic positions on homologous chromosomes or distinct genomic loci.

We identify this as the definitive test of the duplication question and a priority for future work, alongside complete haplotype phasing and functional comparison with *D. setosum*. Detection of structural rearrangements—inversions, translocations, and misjoins—likewise requires a chromosome-scale reference or Hi-C contact data and is therefore deferred to this future work. More broadly, re-annotation of the collapsed reference is the prerequisite for the functional analyses recommended for this system—gene-family expansion and contraction, trait-associated gene characterization, and haplotype-resolved structural-variation analysis—none of which can be performed reliably on the current uncollapsed, allelically duplicated annotation; we identify these as priorities for future work.

### 4.7. Conclusions

The haplotype-resolved genome of *D. antillarum* reported here substantially reduces the haplotype duplication that inflated the initial assembly and provides a near-complete, largely single-copy reference suitable for comparative and conservation genomics. While some genuine segmental duplication or repeat expansions may remain to be characterized with more advanced sequencing technology, the assembly establishes a foundation for understanding the immune biology, population history, and recovery of this ecologically critical Caribbean reef species. More broadly, this study shows that haplotype-aware assembly is essential (not optional) for building accurate reference genomes in highly heterozygous, broadcast-spawning marine invertebrates: it is what collapsed an inflated, doubly represented assembly into a near-complete, largely single-copy reference, and it is on that reference that the immunogenomic, comparative, and conservation studies this keystone species urgently needs can now be built.

## Figures and Tables

**Table 1:** Characteristics of the *D. antillarum* specimens used in this study.

Animal	Collection Site	Coordinates	Collection Date	Tissue	Role in Study
An #1	Rincón, Puerto Rico (west coast)	18°20′35.2″ N, 67°15′40.5″ W	2018	Coelomocytes	ONT reads (gap-patching)
An #2	Rincón, Puerto Rico (west coast)	18°20′35.2″ N, 67°15′40.5″ W	2018	Coelomocytes	ONT reads (gap-patching)
An #3	Punta Escambrón, Puerto Rico (north coast)	18°27′59.70″ N, 66°05′10.26″ W	11 May 2022	Sperm	Reference individual (PacBio HiFi + Illumina)

**Table 2:** Sequencing data generated for the *D. antillarum* genome assembly. ONT data are summarized by sequence yield, as reads were pooled across multiple Flongle and MinION flow cells prior to assembly; PacBio HiFi read-level statistics are reported for the primary assembly data. Coverage is given as raw sequence yield relative to the estimated haploid genome size (~703 Mb); the ~11× value reported in Section 3.5 refers to the modal mapped HiFi depth on the uncollapsed assembly.

Animal	Tissue	Platform	Yield	BioSample	SRA Run	Coverage	Use in Study
An #1	Coelomocytes	ONT (MinION)	4.3 Gb	SAMN46982352	SRR32463885	~6×	Gap-patching (RagTag)
An #2	Coelomocytes	ONT (MinION)	2.6 Gb	SAMN46998589	SRR32479314	~4×	Gap-patching (RagTag)
An #3	Sperm	PacBio HiFi (Sequel IIe)	19.63 Gb (2,878,999 reads; mean 6819 bp; Q34)	SAMN34386196	SRR32365430	~28×	Primary assembly
An #3	Sperm	Illumina MiSeq (2 × 150)	6.1 Gb	SAMN34386197	SRR32478703	~9×	Polishing

**Table 3:** Genome assembly pipelines evaluated in this study. All assemblies used hifiasm (v0.19.8) in default mode. RagTag post-processing differed between pipelines: scaffolding (ordering/orientation against an ONT reference) in v0.3, and patching (gap/missing-region filling) in v0.4; no classical scaffolding was performed in v0.4. The haplotype-resolved assembly was generated with hifiasm haplotype-aware mode (—dual-scaf).

Pipeline	Assembled Reads	RagTag Post-Processing	Racon Polishing
hifiasm v0.1	HiFi	—	—
hifiasm v0.2	HiFi + ONT	—	—
hifiasm v0.3	HiFi	Scaffolding (ONT reference)	—
hifiasm v0.4	HiFi	Patching (ONT reads)	Illumina reads
Haplotype-resolved (—dual-scaf)	HiFi	None	Illumina reads (2 rounds Racon, Minimap2 -ax sr)

**Table 4:** Genome completeness (BUSCO) across assembly stages. Complete (C), single-copy (S), duplicated (D), Fragmented (F), Missing (M). Lineage set is indicated per row; absolute counts across odb10 and odb12 are not directly comparable. Single-copy completeness rises from the default primary (incomplete haplotype collapse) to the haplotype-resolved assemblies.

Assembly	Size (Gb)	Lineage Set (n)	C %	S %	D %	F %	M %
hifiasm v0.4 (initial)	1.75	metazoa_odb10 (954)	98.4	14.0	84.4	1.0	0.6
v0.4 after Purge_Dups	1.24	metazoa_odb10 (954)	97.4	62.5	34.9	1.7	0.9
—dual-scaf primary	1.03	metazoa_odb12 (672)	99.0	85.0	14.0	1.0	0.0
—dual-scaf hap1	0.95	metazoa_odb12 (672)	94.3	88.1	6.2	3.6	2.1
—dual-scaf hap2	0.89	metazoa_odb12 (672)	91.7	90.2	1.5	2.8	5.5

## Data Availability

The initial genome assembly (v0.4) OU_Dant_1.0 is available under GenBank accession GCA_040938485.1 (RefSeq GCF_040938485.1; WGS project JBFRCO000000000), with the corresponding NCBI annotation under GCF_040938485.1-RS_2025_02. The PacBio HiFi reads used to generate the assembly are deposited in the NCBI SRA under run accession SRR32365430. Oxford Nanopore reads (SRR-linked experiments SRX27793263 and SRX27778283) and Illumina reads (SRX27792652) used for gap-patching and polishing are available under BioProject PRJNA839760. The RNA-Seq data generated by our laboratory and used in genome annotation are available under run accessions SRR24973326 and SRR24973327 (gonad, Ion Torrent) and SRR29948272 (esophagus, Illumina). The custom repeat database generated with RepeatModeler v2.0.3 is deposited as SRA run SRR32898058. The custom Python script for reporting statistical results has been deposited on Zenodo (zenodo.15172401, CC0). The haplotype-resolved assemblies (Racon-polished collapsed primary, hap1, and hap2) and the Purge_Dups outputs have been deposited at Zenodo (https://doi.org/10.5281/zenodo.20682733 (accessed on 24 July 2026)) and cross-linked to the bioRxiv preprint (https://doi.org/10.64898/2026.05.24.727502). Sniffles analysis, including the structural-variant call set (VCF), can be found at https://zenodo.org/records/21483215 (accessed on 24 July 2026).

## Supplementary Materials

The following supporting information can be downloaded at: https://www.mdpi.com/article/10.3390/genes17080876/s1, Table S1: Software tools, versions, and source repositories, in approximate order of use across the workflow. Table S2: Comparative assembly and genome metrics for *Diadema antillarum* (this study) and the chromosome-level *Diadema setosum* reference (CUHK_Dset_2.0) [5]. Table S3: Repeat content of the *Diadema antillarum* hifiasm v0.4 assembly compared with the high-coverage read fractions. Repeats were identified with RepeatMasker using a custom RepeatModeler library. The v0.4 column reports the full assembly; the ≥33× and ≥100× columns report HiFi reads mapping to regions above three times the modal coverage (>33×) and above 100×, respectively, extracted for inspection. All repeat values are percent of bases masked. Figure S1: Ploidy assessment of adapter-removed HiFi reads with Smudgeplot, using k-mer sizes 21 (A) and 31 (B). Plots show normalized minor k-mer coverage [B/(A+B), *x*-axis] versus total k-mer-pair coverage (A+B, *y*-axis); color indicates k-mer-pair density. In both panels, the AB genotype predominates (0.61 at k = 21; 0.73 at k = 31), consistent with a normal two-copy (diploid) genome. The subordinate AAB, AAAB, and AABB configurations are most consistent with the high heterozygosity and abundant repetitive content of this genome, producing additional allele-dosage signals, rather than with a higher ploidy level. These minor signals do not, however, exclude the presence of genuine segmental duplication; in highly heterozygous broadcast-spawning invertebrates, k-mer-based dosage signals can reflect hemizygosity and structural variation rather than allelic heterozygosity alone [25], and distinguishing incompletely separated alleles from real duplicated regions would require a higher-quality, chromosome-scale assembly [11]. Figure S2: Bandage assembly graph of the initial (uncollapsed) hifiasm v0.4 assembly (2964 contigs; longest 14,326,224 bp, shortest 12,568 bp). Each contig is drawn as a separate curved line; base-pair lengths omitted for clarity. Figure S3: Length and depth characteristics of large duplicated structural-variant (SV) reads (>1000 bp) in the initial v0.4 assembly. (A) Read-length distribution (log-scale frequency); most reads fall between ~6000 and ~10,000 bp. (B) Coverage-depth distribution for the same reads (log frequency scale), extending to ~80×. These reads represent the residual duplication signal whose origin—incompletely separated alleles versus genuine recent paralogy—cannot be resolved with the present assembly and is identified as a target for future telomere-to-telomere assembly (Section 4.6). Figure S4: Merqury k-mer copy-number spectrum (spectra-cn) for the initial (uncollapsed) hifiasm assembly of *Diadema antillarum*, from a hybrid PacBio HiFi + Illumina 21-mer database. The prominent copy-2 peak, at approximately twice the haploid k-mer multiplicity, reflects retained haplotype duplication in the uncollapsed assembly, consistent with the incomplete-collapse signal described in Section 3.1 and Section 4.1. Reference-free consensus quality QV 44.5; k-mer completeness 97.5%. Figure S5: Structural variants (≥1 kb) called by Sniffles2 (v2.6.0) [23] from a self-alignment of the HiFi reads to the initial (v0.4, uncollapsed) assembly. (A) Variant count by type: 1289 deletions, 388 insertions, 34 duplications, 15 inversions, and 600 breakends. (B) Cumulative genomic span by type (Mb); breakends contribute no span. Deletions, insertions, and duplications together span 9.3 Mb (0.53% of the assembly); inversions add a further 8.6 Mb. Because the reads were aligned to the assembly they built, these calls are predominantly heterozygous, reflecting differences between the two retained haplotypes of the single sequenced individual, with breakends largely reflecting contig fragmentation, rather than external or population-level structural variation (Section 3.5). References [5,10,11,23,25,29,30] are cited in the Supplementary Materials.
