# Supplementary material for: A Haplotype-Resolved Genome Assembly of the Long-Spined Sea Urchin Diadema antillarum, a Keystone Caribbean Reef Herbivore
Source: Genes (Basel). 2026 Jul 28;17(8):876. doi: 10.3390/genes17080876 (PMC13511894; doi:10.3390/genes17080876)
Supplement: Supplementary file 1 [file genes-17-00876-s001.zip › genes-4407900-supplementary.pdf]

Supplementary Materials

A haplotype-resolved genome assembly of the long-spined sea urchin *Diadema antillarum*, a keystone Caribbean reef herbivore

Audrey J. Majeske [1,2,†], Juliet M. Wong [3,4,†], Carlos A. Farkas Pool [5], Jose M. Eirin-Lopez [4], Jose V. Lopez [6], Walter Wolfsberger [1], Nikolaos V. Schizas [7], Alondra M. Díaz-Lameiro [2], Stephanie O. Castro-Márquez [1], Kenneth Hilkert [1], Alejandro J. Mercado Capote [2] and Taras K. Oleksyk [1,2,8,*]

The following supporting information is available online.

Table S1: Software tools, versions, and source repositories.

Table S2: Comparative assembly and genome metrics for *D. antillarum* and *D. setosum*.

Table S3: Repeat content of the hifiasm v0.4 assembly and high-coverage read fractions.

Figure S1: Smudgeplot ploidy assessment.

Figure S2: Bandage assembly graph.

Figure S3: Length and depth characteristics of large, duplicated SV reads.

Figure S4: Merqury k-mer copy-number spectrum of the initial assembly.

Figure S5: Structural-variant counts and genomic span by type (Sniffles2).

Supplementary text — Software and reproducibility

All bioinformatic tools used in this study, with versions and source repositories, are listed in Table S1. Command-line parameters not given in the main text are available in the project repository (Zenodo: https://doi.org/10.5281/zenodo.20682733).

Supplementary Tables

Table S1. Software tools, versions, and source repositories, in approximate order of use across the workflow.

Tool	Version	Source
HiFiAdapterFilt	3.0.0	github.com/sheinasim-USDA/HiFiAdapterFilt
Jellyfish	2.2.10	github.com/gmarcais/Jellyfish
GenomeScope2.0	2.0	github.com/tbenavi1/genomescope2.0
FastK	1.1	github.com/thegenemyers/FASTK
Smudgeplot	0.2.6	github.com/KamilSJaron/smudgeplot
Guppy	6.1.5	community.nanoporetech.com
FastQC	0.12.1	bioinformatics.babraham.ac.uk
Trimmomatic	0.39	usadellab.org
BLAST+	2.16.0	Camacho et al., 2009
hifiasm	0.19.8	github.com/chhylp123/hifiasm
RagTag	2.1.0	github.com/malonge/RagTag
Racon	1.5.0	github.com/lbcb-sci/racon
Minimap2	2.28	github.com/lh3/minimap2
SAMtools	1.21	htslib.org
Sniffles	2.6.0	github.com/fritzsedlazeck/Sniffles
Purge_Dups	1.2.5	github.com/dfguan/purge_dups
RepeatModeler	2.0.3	github.com/Dfam-consortium/RepeatModeler
RepeatMasker	4.1.5	repeatmasker.org
QUAST	5.2.0	quast.sourceforge.net
BUSCO	5.3.0	busco.ezlab.org
Bandage	0.8.1	rrwick.github.io/Bandage
BlobToolKit	3.0.6	blobtoolkit.genomehubs.org
Matplotlib	3.10.1	matplotlib.org
Merqury / meryl	1.4.1	github.com/marbl/merqury
GNU Awk	5.1.0	gnu.org/software/gawk
NCBI EGAP	10.3	ncbi.nlm.nih.gov/genome/annotation_euk
STAR (EGAP)	—	github.com/alexdobin/STAR
Splign / ProSplign (EGAP)	—	Kapustin et al., 2008
WindowMasker (EGAP)	—	Morgulis et al., 2006

Table S2. Comparative assembly and genome metrics for *Diadema antillarum* (this study) and the chromosome-level *Diadema setosum* reference (CUHK_Dset_2.0; Hong Kong Biodiversity Genomics Consortium et al., 2024).

Metric	*D. antillarum* initial (uncollapsed)	*D. antillarum* primary (collapsed reference)	*D. antillarum* hap1	*D. antillarum* hap2	*D. setosum* (CUHK_Dset_2.0)
Role in study	Initial assembly	**Recommended reference**	Haplotype 1	Haplotype 2	Congener reference
Assembly size	1.75 Gb (1,754,659,819 bp)	1.03 Gb (1,028,719,238 bp)	0.95 Gb (946,558,832 bp)	0.89 Gb (892,201,912 bp)	885.8 Mb (885,842,048 bp)
Scaffolds	n/a	n/a	n/a	n/a	101
Contigs	2,964	2,051	2,860	2,400	513
Scaffold N50	—	—	—	—	38.3 Mb (L50 = 11)
Contig N50	1.57 Mb	1.20 Mb	662 kb	682 kb	3.5 Mb (L50 = 84)
Largest contig	14.3 Mb	5.42 Mb	5.41 Mb	4.98 Mb	52.8 Mb (scaffold)
Chromosomes	none (contig-level)	none	none	none	22 (2n = 44; 98.6% anchored)
GC content	38.5%	38.43%	36.98% ‡	37.03% ‡	38.5%
BUSCO complete (C)	98.4%	99.0%	94.3%	91.7%	98.1%
— single-copy (S)	14.0%	85.0%	88.1%	90.2%	97.8%
— duplicated (D)	84.4%	14.0%	6.2%	1.5%	0.3%
— fragmented (F)	1.0%	1.0%	3.6%	2.8%	1.2%
— missing (M)	0.6%	0.0% §	2.1% §	5.5% §	0.7%
BUSCO lineage	metazoa_odb10	metazoa_odb12	metazoa_odb12	metazoa_odb12	metazoa_odb10
Heterozygosity (k-mer)	2.52%	2.52%	2.52%	2.52%	2.11% (2.07–2.15%)
Repeat content	42.84% (29.96% unclassified)	—	—	—	—
Protein-coding genes	33,123 (EGAP)	not re-annotated	—	—	23,030 (27,478 gene models)
Sequencing	PacBio HiFi 19.6 Gb, ~28×	(same data)	(same data)	(same data)	PacBio HiFi 18.5 Gb, ~21× + Omni-C
Accession / deposit	GCA_040938485.1	Zenodo 10.5281/zenodo.20682733	Zenodo (same)	Zenodo (same)	GCA_033980235.1

Footnotes:

† The v0.4 assembly is the initial uncollapsed assembly and is not the deliverable.

‡ The collapsed primary reference was polished with two rounds of Racon (Illumina reads); hap1 and hap2 are reported pre-polishing. The ~1.4-point GC difference between the polished primary (38.43%) and the unpolished haplotypes (~37%) reflects this polishing step.

§ Missing (M) for the dual-scaf assemblies derived as 100 − C − F; confirm against the raw BUSCO summary. Heterozygosity is a species-level estimate (GenomeScope2, k = 21) and applies to all *D. antillarum* assemblies. *D. setosum* values are from Hong Kong Biodiversity Genomics Consortium et al., 2024 (GigaByte, doi:10.46471/gigabyte.121); BUSCO is genome-mode metazoa_odb10, directly comparable to *D. antillarum* v0.4 (also odb10) but not strictly to the collapsed primary/haplotype assemblies (odb12).

Table S3. Repeat content of the Diadema antillarum hifiasm v0.4 assembly compared with the high-coverage read fractions. Repeats were identified with RepeatMasker using a custom RepeatModeler library. The v0.4 column reports the full assembly; the ≥33× and ≥100× columns report HiFi reads mapping to regions above three times the modal coverage (>33×) and above 100×, respectively, extracted for inspection. All repeat values are percent of bases masked.

Repeat class	v0.4 assembly	≥33× reads	≥100× reads
Count	2,964 contigs	53,387 reads	7,132 reads
Total length (bp)	1,754,659,819	373,089,082	49,529,407
GC level (%)	38.48	39.69	48.90
Bases masked (%)	42.84	61.52	93.07
Retroelements	6.15	13.62	49.43
SINEs	0.62	0	0
LINEs	3.86	12.61	49.43
LTR elements	1.67	1.01	0
DNA transposons	3.09	0.74	0
Rolling-circles	0.09	1.67	0
Unclassified	29.96	42.25	41.46
Total interspersed repeats	39.68	56.62	90.89
Small RNA	0.58	0.03	0
Satellites	0.02	0	0
Simple repeats	2.49	2.67	2.07
Low complexity	0.36	0.53	0.11

Supplementary figures

Figure S1. Ploidy assessment of adapter-removed HiFi reads with Smudgeplot, using k-mer sizes 21 (A) and 31 (B). Plots show normalized minor k-mer coverage [B/(A+B), x-axis] versus total k-mer-pair coverage (A+B, y-axis); color indicates k-mer-pair density. In both panels, the AB genotype predominates (0.61 at k = 21; 0.73 at k = 31), consistent with a normal two-copy (diploid) genome. The subordinate AAB, AAAB, and AABB configurations are most consistent with the high heterozygosity and abundant repetitive content of this genome, producing additional allele-dosage signals, rather than with a higher ploidy level. These minor signals do not, however, exclude the presence of genuine segmental duplication; in highly heterozygous broadcast-spawning invertebrates, k-mer-based dosage signals can reflect hemizygosity and structural variation rather than allelic heterozygosity alone (Calcino et al., 2021), and distinguishing incompletely separated alleles from real duplicated regions would require a higher-quality, chromosome-scale assembly (see Section 4.6; Ranallo-Benavidez et al., 2020).

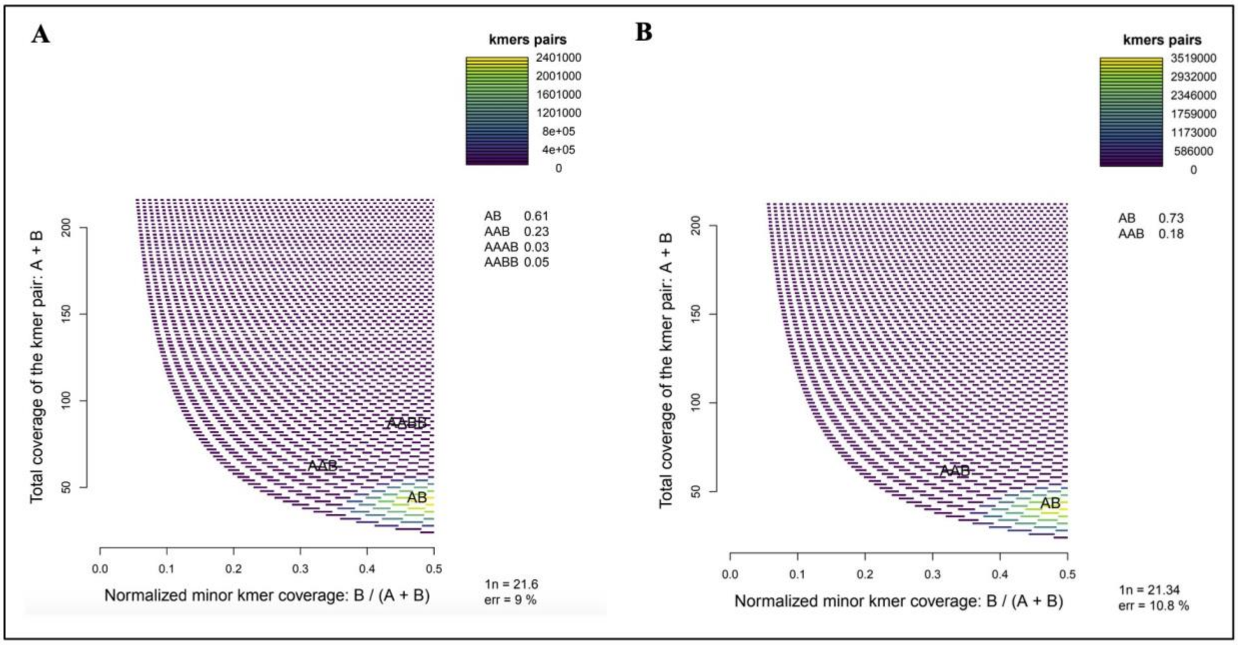

Figure S2. Bandage assembly graph of the initial (uncollapsed) hifiasm v0.4 assembly (2,964 contigs; longest 14,326,224 bp, shortest 12,568 bp). Each contig is drawn as a separate curved line; base-pair lengths omitted for clarity.

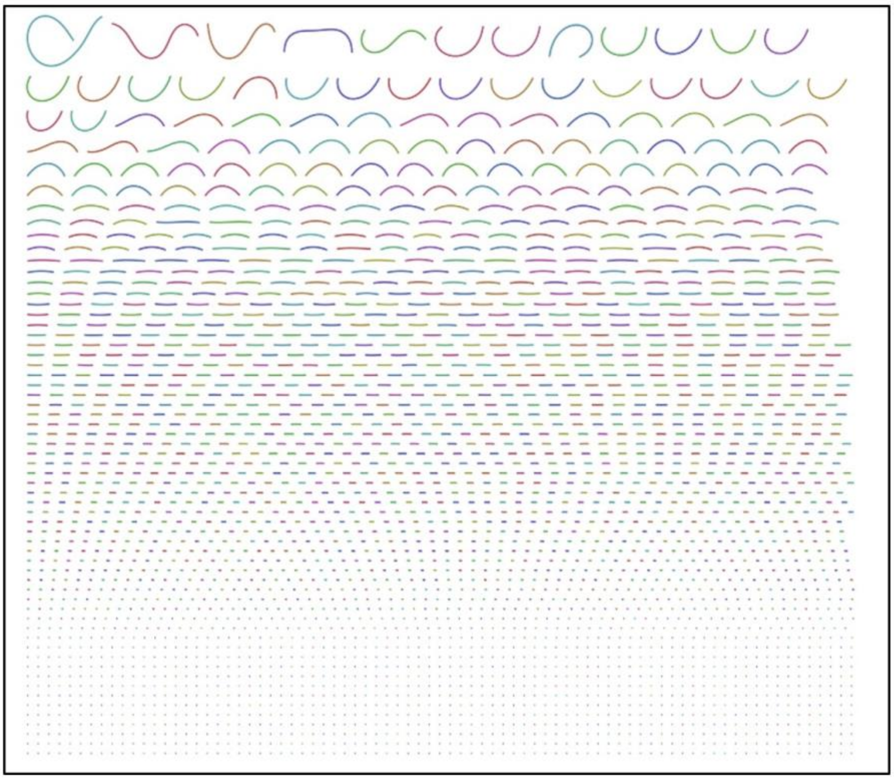

Figure S3. Length and depth characteristics of large duplicated structural-variant (SV) reads (>1000 bp) in the initial v0.4 assembly. (A) Read-length distribution (log-scale frequency); most reads fall between ~6,000 and ~10,000 bp. (B) Coverage-depth distribution for the same reads (log frequency scale), extending to ~80×. These reads represent the residual duplication signal whose origin — incompletely separated alleles versus genuine recent paralogy — cannot be resolved with the present assembly and is identified as a target for future telomere-to-telomere assembly (Section 4.6).

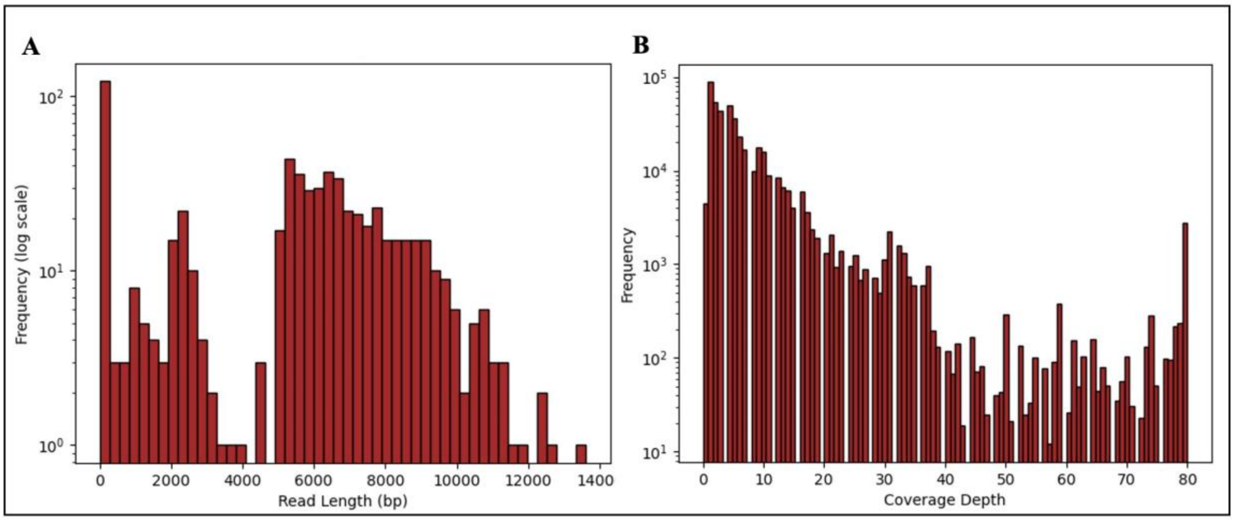

Figure S4. Merqury k-mer copy-number spectrum (spectra-cn) for the initial (uncollapsed) hifiasm assembly of *Diadema antillarum*, from a hybrid PacBio HiFi + Illumina 21-mer database. The prominent copy-2 peak, at approximately twice the haploid k-mer multiplicity, reflects retained haplotype duplication in the uncollapsed assembly — consistent with the incomplete-collapse signal described in Sections 3.1 and 4.1. Reference-free consensus quality QV 44.5; k-mer completeness 97.5%.

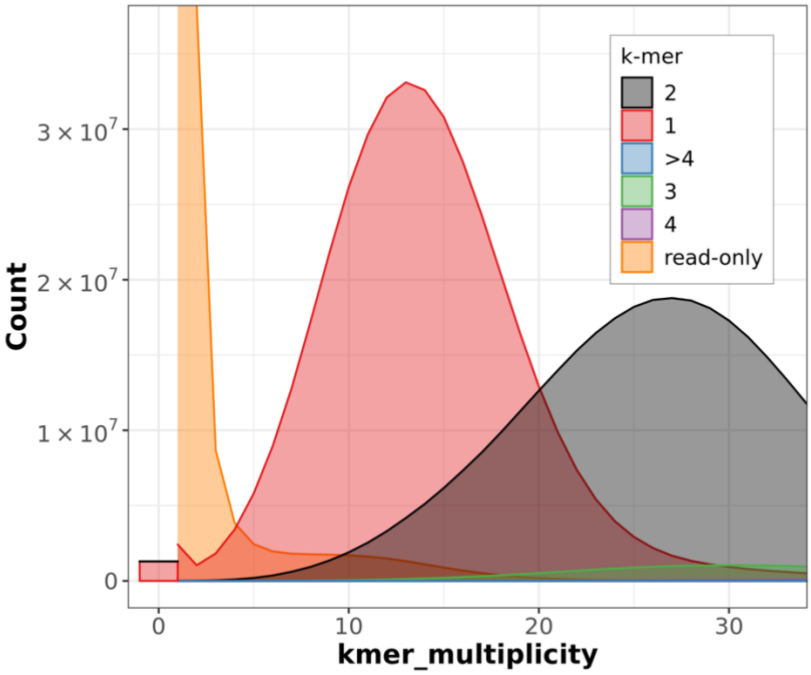

Figure S5. Structural variants (≥ 1 kb) called by Sniffles2 (v2.6.0) from a self-alignment of the HiFi reads to the initial (v0.4, uncollapsed) assembly. (A) Variant count by type: 1,289 deletions, 388 insertions, 34 duplications, 15 inversions, and 600 breakends. (B) Cumulative genomic span by type (Mb); breakends contribute no span. Deletions, insertions, and duplications together span 9.3 Mb (0.53% of the assembly); inversions add a further 8.6 Mb. Because the reads were aligned to the assembly they built, these calls are predominantly heterozygous — reflecting differences between the two retained haplotypes of the single sequenced individual, with breakends largely reflecting contig fragmentation — rather than external or population-level structural variation (Section 3.5).

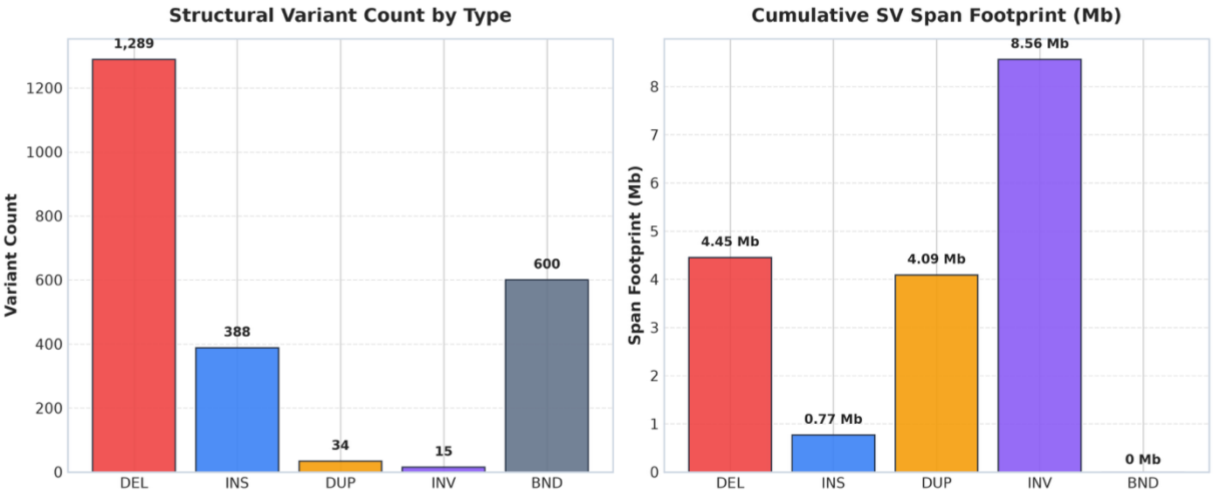

References cited in the Supplementary Materials

Calcino, A.D.; Kenny, N.J.; Gerdol, M. Single individual structural variant detection uncovers widespread hemizygosity in molluscs. Philos. Trans. R. Soc. B Biol. Sci. 2021, 376, 20200153. https://doi.org/10.1098/rstb.2020.0153

Camacho, C.; Coulouris, G.; Avagyan, V.; Ma, N.; Papadopoulos, J.; Bealer, K.; Madden, T.L. BLAST+: Architecture and applications. BMC Bioinform. 2009, 10, 421. https://doi.org/10.1186/1471-2105-10-421

Hong Kong Biodiversity Genomics Consortium; Hui, J.H.L.; Chan, T.F.; et al. Chromosomal-level genome assembly of the long-spined sea urchin Diadema setosum (Leske, 1778). GigaByte 2024. https://doi.org/10.46471/gigabyte.121

Kapustin, Y.; Souvorov, A.; Tatusova, T.; Lipman, D. Splign: Algorithms for computing spliced alignments with identification of paralogs. Biol. Direct 2008, 3, 20. https://doi.org/10.1186/1745-6150-3-20

Morgulis, A.; Gertz, E.M.; Schäffer, A.A.; Agarwala, R. WindowMasker: Window-based masker for sequenced genomes. Bioinformatics 2006, 22, 134–141. https://doi.org/10.1093/bioinformatics/bti774

Ranallo-Benavidez, T.R.; Jaron, K.S.; Schatz, M.C. GenomeScope 2.0 and Smudgeplot for reference-free profiling of polyploid genomes. Nat. Commun. 2020, 11, 1432. https://doi.org/10.1038/s41467-020-14998-3
